# Molecular xenosurveillance of *Aedes* mosquitoes reveals dengue virus serotype-2 during an outbreak in Dhaka, Bangladesh

**DOI:** 10.1128/spectrum.01769-25

**Published:** 2025-09-26

**Authors:** Prakash Ghosh, Anupama Hazarika, Md. Arko Ayon Chowdhury, Sabera Sultana, Nishad Tasnim Mithila, Shariful Shahid, Md Ekramul Haque, Md Nazmul Islam, Dinesh Mondal, Rajib Chowdhury

**Affiliations:** 1 Maternal and Child Nutrition, Nutrition Research Division, icddr,b 56291, Dhaka, Bangladesh; 2 Institute for Animal Hygiene and Veterinary Public Health, Leipzig University 9180, Leipzig, Germany; 3 Communicable Disease Surveillance (CDS), World Health Organization Bangladesh 215134, Dhaka, Bangladesh; 4 Communicable Disease Control Unit, Directorate General of Health Services (DGHS), Dhaka, Bangladesh; 5 Local Government Division, Ministry of Local Government, Rural Development and Co-operatives 316327 , Dhaka, Bangladesh; Connecticut Agricultural Experiment Station, New Haven, Connecticut, USA

**Keywords:** dengue, molecular xenosurveillance, *Aedes aegypti*, outbreak, Bangladesh

## Abstract

The increased risk of emerging infectious diseases is particularly pronounced in resource-constrained regions with limited capacity to undertake surveillance, presenting a global challenge for the timely identification and mitigation of disease threats. Consequently, the development and implementation of feasible, cost-effective, and sustainable surveillance strategies are essential. Molecular xenosurveillance (MX) represents an innovative approach for arbovirus monitoring, including dengue surveillance, which utilizes field-collected mosquito specimens to detect circulating pathogens and provide early outbreak warnings. Bangladesh is highly endemic for dengue, and with limited resources, the implementation of MX will add value to ongoing efforts to scale up surveillance. This study aimed to test the suitability of MX in detecting dengue viruses in *Aedes* vectors and recommend its integration into the existing surveillance system. Adult *Aedes* mosquitoes were collected using BG-Sentinel traps during pre-monsoon dengue vector surveys in Dhaka city. *Aedes* mosquitoes were identified morphologically and screened in pools using real-time RT-PCR. Infection prevalence was estimated using the PoolTestR program. Out of 228 *Aedes aegypti* mosquitoes distributed across 26 pools, one pool with 10 mosquitoes tested positive for the dengue virus. The estimated infection prevalence was 0.450% (95% CI: 0.026%–1.960%) and 0.510% (95% CI: 0.053%–2.100%) using maximum likelihood and Bayesian estimates, respectively. The DENV-2 serotype was detected in the positive pool. This study presents the first successful implementation of the MX methodology to detect dengue virus presence in field-collected *Ae. aegypti* specimens from Dhaka, Bangladesh. These findings provide empirical support for enhanced vector surveillance protocols and offer valuable insights for strengthening comprehensive dengue control efforts.

**IMPORTANCE:**

The timely detection of arboviral pathogens like dengue virus, through surveillance is critical for developing early warning systems and preventing outbreaks. However, limited surveillance capacity in resource-constrained settings creates gaps that can be addressed by implementing sustainable and cost-effective surveillance strategies. This study demonstrates the feasibility of molecular xenosurveillance (MX), which is a vector-based, non-invasive surveillance method for dengue virus surveillance in *Aedes aegypti* mosquitoes in Dhaka, Bangladesh. By detecting and identifying dengue virus serotype-2 (DENV-2) using real-time RT-PCR, this study also provides evidence of the method's potential for generating deeper epidemiological insights. These findings highlight the value of MX as a complementary tool to existing surveillance systems. Integrating MX into routine entomological surveillance could significantly enhance national preparedness and response to dengue outbreaks.

## INTRODUCTION

The World Health Organization (WHO) estimated that 80% of the worldwide populace is vulnerable to one or more vector-borne diseases (VBDs), contributing nearly 17% to the overall burden of communicable diseases (1). Meanwhile, the Southeast Asian region has become the epicenter for emerging infectious diseases (EIDs), particularly VBDs having epidemic or pandemic potential (2). Vital factors, including population growth and high density, improper urbanization, mobility, and shifts in the environment, have a substantial impact on the emergence of infectious diseases (1).

Dengue virus infection is one of the VBDs primarily transmitted by infectious female *Aedes* mosquitoes. The WHO has flagged dengue as one of the top ten global health concerns (3). Over the past two decades, since 2000, the number of reported dengue cases increased 10-fold worldwide, rising from 500,000 to 5.2 million (4).

The earliest recorded dengue case in Bangladesh dates back to 1964. The illness was informally termed “Dacca fever” at that time. However, the disease did not draw attention as a major health problem until a major outbreak happened in 1999 at Dhaka, followed by an epidemic in 2000 (5, 6). Since then, Dhaka and other metropolitan cities have been facing the scourge of dengue outbreaks periodically, with Dhaka being the epicenter. The Directorate General of Health Services (DGHS), Bangladesh, officially reported 101,354, 28,429, 61,763, 321,179, and 101,214 hospitalized dengue cases annually in 2019, 2021, 2022, 2023, and 2024, respectively (7–10). Among the four dengue virus serotypes, DENV-1 and DENV-2 were prevalent between 2013 and 2016, whereas in the outbreak in 2019, DENV-3 serotype was the most prevalent one in Bangladesh (7). During the outbreak in 2023, the serotype DENV-2 reappeared as the predominant serotype (11).

In the absence of established therapeutic or preventive intervention, effective strategies for controlling and minimizing transmission revolve around implementing vector control measures and enhancing public awareness. WHO has denoted vector surveillance, monitoring, and evaluation of interventions as one of the four pillars to achieve effective and sustainable vector control (1). In recent years, the notion of establishing an early warning system has been proposed in various countries as an approach to contain dengue transmission (12–15). Developing an effective early warning system to predict outbreaks of dengue is sophisticated as it involves several interacting factors including host, environment, vector, and dengue virus itself. Therefore, indicators spanning meteorological, epidemiological, population, and entomological (both vector abundance and infection) are essential for establishing a robust early warning system. Xenosurveillance, which involves the use of blood-fed hematophagous arthropods to monitor and identify vertebrate pathogens (16, 17), offers a valuable approach in this context. Tracking dengue viruses in both immature and adult mosquitoes is a pivotal strategy for detecting the onset of the epidemic phase and guiding timely measures (18).

Previous entomological studies on dengue in Bangladesh have mostly concentrated on assessing vector density and distribution. However, estimating and mapping the prevalence of dengue virus in mosquitoes are also essential (19, 20), as mentioned earlier. Successful implementation of a vector control program necessitates comprehensive information about the vectors, encompassing their density, resistance to control methods, and infection rates (21). Despite the high dengue burden and risk of transmission in Bangladesh, especially in Dhaka, there is inadequate data on the vector infection rate. Low socio-economic regions of Dhaka were found to be abundant with *Aedes* mosquitoes, but their infection with dengue viruses has not been explored (20).

Molecular xenosurveillance or xenomonitoring (MX) is a modern tool to analyze genetic material (DNA or RNA) from field-derived vectors to detect the presence of pathogens for disease surveillance. It has emerged as a potential surveillance technique due to substantial advancements in laboratory techniques. MX offers a sensitive and tractable approach for tracking pathogen circulation in the vector population even before in the human population, making it a valuable tool for early outbreak detection and response. Periodical MX has successfully been applied as a post-mass drug administration surveillance tool for global programs to eliminate lymphatic filariasis (22, 23). Several countries endemic to dengue are now putting efforts into establishing MX for assessing the prevalence of dengue infection in vectors, identifying transmission hotspots, predicting outbreaks through predictive modeling, and eventually, devising comprehensive early warning systems to implement timely control measures (18, 24–26). This innovative approach has the potential to revolutionize VBD/EID surveillance and is seemingly fit for programmatic use in Bangladesh (22). Thus, we have introduced xenosurveillance for the first time in Dhaka city for detecting and identifying dengue virus in *Aedes aegypti* vectors. The study aimed to establish MX for the dengue in settings like Bangladesh, thereby strengthening existing entomological surveillance and guiding large-scale implementation in areas prone to dengue outbreaks.

## RESULTS

### Vector distribution and physiological status

A total of 1,350 mosquitoes were collected (Table 1) from the 26 traps (6 days collection period) where the number of *Ae. aegypti*, *Culex quinquefasciatus, Culex tritaeniorhynchus,* and *Armigeres subalbatus* were 228, 992, 104, and 26, respectively. *Cx. quinquefasciatus* was the most abundant species, accounting for 73.48% of total captures, significantly exceeding the expected proportion under an equal-distribution assumption (χ² = 1,753.8, df =3, *P* < 0.001). The other three species *Ae. aegypti* (16.89%), *Cx. tritaeniorhynchus* (7.70%), and *Ar. subalbatus* (1.93%) were significantly under-represented compared to *Cx. quinquefasciatus*, with *Ar. subalbatus* being particularly rare.

**TABLE 1:** Abundance and sex distribution of field-collected mosquito species by study sites along with the physiological status of female *Ae. aegypti*

Study site	Species	Number collected (*N*)	Male (*N*)	Female (*N*)	Physiological status of female *Ae. aegypti*			Total
					Fed	Unfed	Gravid	
Rampura	*Ae. aegypti*	20	6	14	6	8	0	85
	*Cx. quinquefasciatus*	51	30	21	–^*a*^	–	–	
	*Cx. tritaeniorhynchus*	10	5	5	–	–	–	
	*Ar. subalbatus*	4	1	3	–	–	–	
Basabo	*Ae. aegypti*	62	21	41	13	23	5	796
	*Cx. quinquefasciatus*	684	394	290	–	–	–	
	*Cx. tritaeniorhynchus*	46	28	18	–	–	–	
	*Ar. subalbatus*	4	3	1	–	–	–	
Kamalapur	*Ae. aegypti*	71	26	45	15	23	7	152
	*Cx. quinquefasciatus*	71	40	31	–	–	–	
	*Cx. tritaeniorhynchus*	10	6	4	–	–	–	
	*Ar. subalbatus*	0	0	0	–	–	–	
Mirpur	*Aee. aegypti*	25	9	16	6	9	1	210
	*Cx. quinquefasciatus*	157	91	66	–	–	–	
	*Cx. tritaeniorhynchus*	25	16	9	–	–	–	
	*Ar. subalbatus*	3	2	1	–	–	–	
Banani	*Ae. aegypti*	19	5	14	4	8	2	36
	*Cx. quinquefasciatus*	12	7	5	–	–	–	
	*Cx. tritaeniorhynchus*	5	4	1	–	–	–	
	*Ar. subalbatus*	0	0	0	–	–	–	
Dhanmondi	*Ae. aegypti*	16	4	12	4	5	3	56
	*Cx. quinquefasciatus*	17	13	4	–	–	–	
	*Cx. tritaeniorhynchus*	8	5	3	–	–	–	
	*Ar. subalbatus*	15	10	5	–	–	–	
Baunia	*Ae. aegypti*	15	9	6	0	6	0	15
	*Cx. quinquefasciatus*	0	0	0	–	–	–	
	*Cx. tritaeniorhynchus*	0	0	0	–	–	–	
	*Ar. subalbatus*	0	0	0	–	–	–	
	**Total**	**1,350**	**735**	**615**	**48**	**82**	**18**	

^*a*^ “–” indicates that data were not collected because it was outside the scope of interest.

Among the collected *Ae. aegypti*, 148 and 80 were female and male, respectively. In female *Ae. aegypti* population*,* 48, 82, and 18 were fed, unfed, and gravid, respectively (Table 1). During the collection period, two traps had only *Ae. aegypti*, one trap had only *Culex* spp., and in seven traps, no mosquito was trapped. The captured *Ae. aegypti* (both female and male) were further distributed in 26 pools based on the trapping sites.

### Molecular screening of the pools and prevalence estimation

Out of these 26 pools, one pool from Basabo area containing 10 *Ae. aegypti* mosquitoes tested positive for the dengue virus in pan-dengue RT-qPCR screening. Upon further examination utilizing RT-PCR, the virus within the positive pool was confirmed as a DENV-2 serotype. Using the PoolPrev function of the PoolTestR tool, the PCR results were analyzed considering the entire data set from a single study region (Dhaka City). The maximum likelihood estimate indicated a dengue infection prevalence of 0.450% (95% CI: 0.026%–1.960%). On the other hand, the Bayesian estimation suggested a slightly higher prevalence rate of 0.51% (95% CI: 0.053%–2.100%). The Minimum Infection Rate (MIR) was determined to be 4.39, considering only one dengue-infected mosquito in the positive pool.

## DISCUSSION

MX has been well acknowledged by the scientific community for the purpose of VBDs/ EIDs surveillance and monitoring. This tool complements vector management strategies and has the potential to alert authorities in advance of outbreaks. However, this technique is yet to be integrated into the ongoing vector surveillance in Bangladesh. Therefore, the overarching goal of this study was to establish a MX tool for the detection and identification of dengue virus in vector mosquitoes.

This study provides the very first evidence of dengue infection in adult *Aedes* mosquitoes detected through MX in Bangladesh. The presence of the infection in the vectors denotes the ongoing transmission of dengue virus in the community. The occurrence of the outbreak during the study further bolsters the findings of the current study and the transmission.

The earlier entomological surveillance studies conducted in Bangladesh were confined to estimating house index (percentage of houses positive with larvae/pupae), container index (percentage of water holding containers positive with larvae/pupae), Breteau index (number of containers positive with larvae/pupae per 100 inspected houses), adult vector abundance, breeding sites, and insecticide resistance status (19, 20, 27–29). Since the abundance does not proportionately represent the vector infection rate and some earlier studies failed to correlate the mentioned indices with the number of cases and the transmission of virus from host to vector, therefore, it is critical to estimate the vector infection rate (30, 31).

In this study, we confirmed the existence of the dengue virus in the vector and distinguished its serotype as DENV-2, which is similar to the predominant serotype (68.1%) found in the patients during the outbreak of 2023 in Bangladesh (32). Serotyping the dengue virus in vectors offers valuable insights into the concurrent circulation of serotypes, thereby complementing the prediction of the magnitude of outbreaks and disease severity (33).

Several dengue-endemic countries have already introduced MX to aid vector-based surveillance. In India, in an endemic urban area, the minimum infection rate was found to be 4.5, which is similar to the minimum infection rate in this study (26). The same study reported one pool of male *Ae. aegypti* to be tested positive for DENV, indicating the importance of including male mosquitoes. Although male mosquitoes do not blood-feed and are not direct vectors, the presence of viral RNA in males may indicate vertical (transovarial) transmission from infected female mosquitoes to their offspring. This mechanism could play a role in maintaining the virus within mosquito populations during inter-epidemic periods and justifies our study to include male mosquitoes for testing. A study conducted in Brazil reported a minimum infection rate of 19.8, where adult *Aedes* vectors were collected for 3 years (18).

An important constraint in this study is the relatively small sample size. Additionally, the sample collection period was very short, which limits the exploration of the seasonal variation of the infectivity rate. Thus, to employ the insights from xenosurveillance in the development of a successful early warning system, a substantial quantity of vectors should be collected year-round and tested for the disease-causing pathogen. Despite proper pool screening methods being used in this study, the observed infection rate does not truly represent Dhaka city. This can be overcome by increasing spatial and temporal coverage (34).

Although this pilot study has limitations, the findings of the study will help in tailoring a robust dengue surveillance strategy. Forecasting dengue outbreaks only through short-term infection surveillance is almost impossible. Thus, year-round surveillance programs are required to provide more precise and accurate insights. Moreover, the inclusion of infection surveillance in the immature mosquitoes (larvae and pupae) to the activity is crucially important for endemic settings to evaluate the conservation of the virus during an inter-epidemic period (35). This tool also has the potential to be used as a proxy to host infection estimation for vector-borne disease surveillance in the future. However, a comprehensive understanding of the correlation between pathogen-infection rates in both humans and vectors is indispensable (22). In addition to disease surveillance, data from MX can aid in formulating or determining the effectiveness of vector control interventions. In Indonesia, the vector infection data derived from MX promises to serve as baseline information for Wolbachia-mediated vector control strategies (31). In addition to the detection of a single pathogen, multiplex molecular assay can be integrated into MX for the detection of multiple pathogens transmitted by the same vector. It is worth noting that dengue, chikungunya, and zika viruses share the same vector; therefore, MX of *Aedes* might provide insights into the combined prevalence of these pathogens or the emergence of a pathogen in a particular area. The current MX tool demands cost- and expertise-intensive methods. Hence, to render MX more cost-effective and feasible, it is imperative to devise a rapid nucleic acid extraction method and simpler molecular assays (RT-RPA, RT-LAMP) alternative to RT-PCR without compromising sensitivity and specificity.

### Conclusion

This study demonstrates the potential of MX to detect dengue virus prevalence in vector mosquitoes and provide valuable epidemiological insights, both of which are critical components for developing robust early warning systems. Furthermore, large-scale prospective studies are essential to optimize the tool towards its integration into existing disease surveillance programs.

## MATERIALS AND METHODS

### Field collection

**Fig 1:**
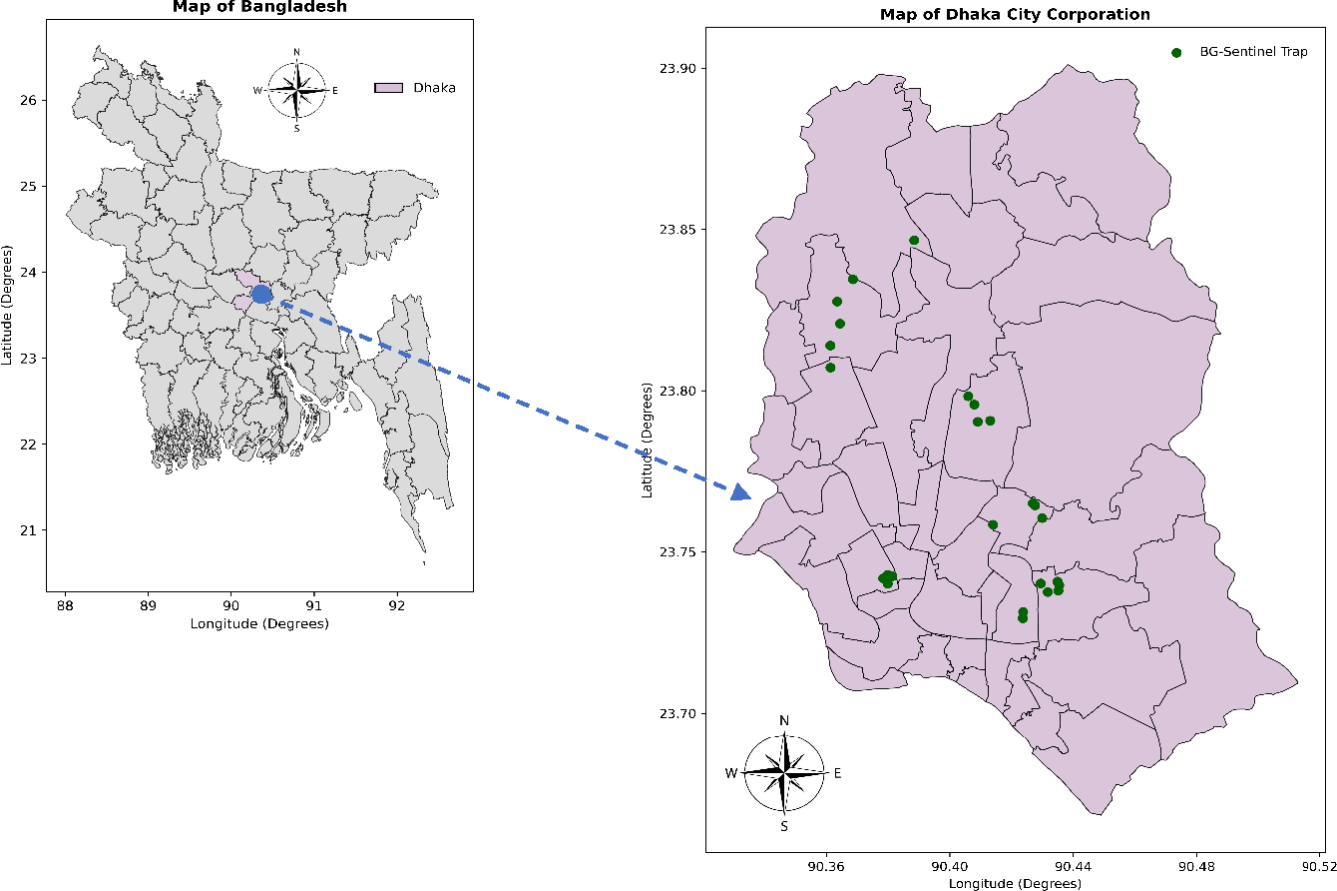
Map indicating the locations of BG-Sentinel two traps used in this study within DNCC and DSCC. The maps were created for data visualization using Python (version 3.12.0) and the shapefile available at https://data.humdata.org/dataset/cod-ab-bgd.

**Fig 2:**
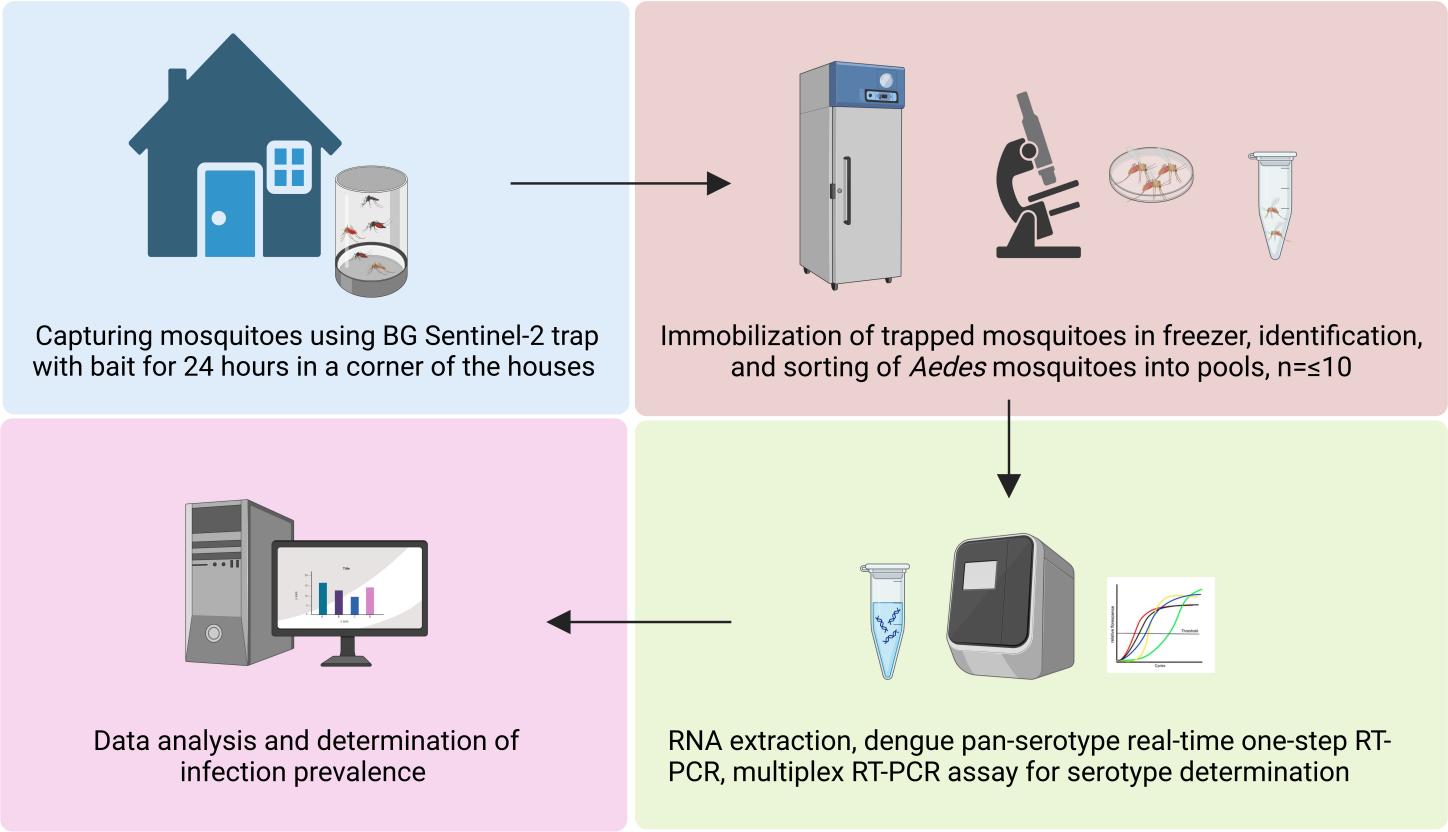
Dengue MX workflow of the study (created with Biorender.com).

As part of a routine pre-monsoon dengue vector survey by the entomological team of the Communicable Disease Control Unit, DGHS, Government of Bangladesh, this cross-sectional study was conducted. Thus, adult mosquitoes were collected from different areas in Dhaka, Bangladesh, by trained entomologists and entomological technicians. Here, BG Sentinel-2 traps were used with 1 tablespoon of yeast with 15 mL of 10% sugar as bait. The areas encompassed Rampura, Basabo, Kamalapur, Mirpur, Baunia, Banani, and Dhanmondi within the Dhaka North City Corporation (DNCC) and Dhaka South City Corporation (DSCC) (Fig. 1). A total of 26 traps were set up in the randomly selected houses between 20–25 June 2023. Each trap was set up in a corner of the house for 24 h (09:00 AM–09:00 AM). The activities carried out to accomplish the study are shown in Fig. 2.

### Mosquito identification and processing

After collecting, the mosquitoes were placed in a −20°C freezer for at least 20 min, within 2 h to immobilize the mosquitoes. Afterward, the adult *Ae. aegypti* mosquitoes were separated from the blend and subjected to morphological speciation and physiological status determination. Mosquito specimens were then pooled based on traps with a number no greater than 10 and labeled accordingly. The pools were then stored at −80°C until the RNA was extracted.

### RNA extraction

RNA from the mosquito pools was extracted employing Qiagen RNeasy Mini Kit. Buffer RLT (600 µL) was added to each tube followed by homogenization using a handheld mini homogenizer with plastic micropistilles until finely homogenized. Ethanol (800 µL; 70%) was added and thoroughly mixed by pipetting. Subsequently, 700 µL of the blend was shifted to an RNeasy spin column and centrifuged at 8,000 × *g* for 15 seconds and the flow-through was disposed of. The remaining blend is put in the same column and the step is repeated. After introducing the samples into the RNeasy Mini spin columns, we adhered to the manufacturer’s guidelines for the subsequent stages in the RNeasy Mini kit protocol. This included applying 700 µL of Buffer RW1, followed by 500 µL of Buffer RPE for each of the two consecutive wash steps. RNA was eluted using 40 µL RNase-free water.

### Real-time one-step RT-PCR

Dengue pan-serotype real-time one-step RT-PCR was performed on RNA extracted from every mosquito pool. The primers and probes used were derived from the work of Leparc-Goffarf et al. (36) and synthesized by Integrated DNA Technologies (IDT). The single-plex RT-PCR was performed on an Applied Biosystems 7500 real-time PCR instrument using 2 µL of extracted RNA, 10 µL of 2× QuantiNova Probe RT-PCR Master Mix, 0.2 µL of Probe RT Mix, 0.1 µL of ROX reference dye, 450 nM of forward and reverse primers, and 225 nM of probes (Taq), with the final volume adjusted to 20 µL using nuclease-free water. The thermal cycling conditions were as follows: 45°C for 12 min, 95°C for 5 min, followed by 40 cycles of 95°C for 5 seconds and 60°C for 40 seconds, while fluorescence data were collected at 60°C.

The RNA from dengue-positive pool was subsequently analyzed using the U.S. Food and Drug Administration (FDA) approved CDC DENV-1-4 Real-Time RT-PCR multiplex assay (package insert, catalog number KK0128; available at https://www.cdc.gov/dengue) to determine the dengue virus serotype (11). The multiplex RT-PCR was performed on a Bio-Rad CFX96 Real-Time PCR Detection System using 5 µL of extracted RNA and along with oligonucleotide primers and fluorescent probes (TaqMan). The thermal cycling conditions were as follows: 50°C for 30 min, 95°C for 2 min, followed by 40 cycles of 95°C for 15 seconds and 60°C for 1 min. Fluorescence data were collected at 60°C. A sample was considered positive if the amplification curve had a cycle threshold (Ct) ≤37, while Ct values >37 were considered negative. In each of the RT-PCR run, nuclease-free water was used as the negative control and molecular standards were used as positive controls.

### Data analysis

Mosquito specimen collection information along, with their distribution in the pool, was entered into a single database. The corresponding molecular screening results from RT-PCR were analyzed using the 7500 Fast Software v2.3 and incorporated in the above-mentioned database. The prevalence of the infection was estimated using the PoolTestR program (with default parameters). It is an R language (R Core Team, 2020) based package for analyzing data from complex MX surveys involving testing of pooled or grouped samples (26). The minimum Infection Rate (MIR) was also determined by dividing the number of DENV-positive pools by the total number of mosquitoes tested and then multiplying by 1000 (18).

## Data Availability

All relevant data are within the paper itself. This study did not involve generating high-throughput sequencing data or other large data sets typically deposited in online repositories.
